# Posterior Intravaginal Slingplasty versus Unilateral Sacrospinous Ligament Fixation in Treatment of Vaginal Vault Prolapse

**DOI:** 10.1155/2013/958670

**Published:** 2013-08-13

**Authors:** Virva Nyyssönen, Anne Talvensaari-Mattila, Markku Santala

**Affiliations:** ^1^Department of Obstetrics and Gynecology, North-Carelian Central Hospital, Tikkamäentie 16, 80210 Joensuu, Finland; ^2^Department of Obstetrics and Gynecology, Oulu University Hospital, P.O. Box 5000, 90014 Oulu, Finland

## Abstract

*Objective*. To investigate the differences in efficacy, postoperative complications, and patient satisfaction between posterior intravaginal slingplasty (PIVS) and unilateral sacrospinous ligament fixation (SSLF) procedures. *Study Design*. A retrospective study of thirty-three women who underwent PIVS or SSLF treatment for vaginal vault prolapse in Oulu University Hospital. The patients were invited to a follow-up visit to evaluate the objective and subjective outcomes. Median follow-up time was 16 months (range 6–52). The anatomical outcome was detected by the Pelvic Organ Prolapse Quantification (POP-Q) system. Information on urinary, bowel, and sexual dysfunctions and overall satisfaction was gathered with specific questionnaire. The data were analyzed using Mann-Whitney *U* test and Fisher's exact test. *Results*. Mesh erosion was found in 4 (25%) patients in the PIVS group. Anatomical stage II prolapse or worse (any POP-Q point ≥−1) was detected in 8 (50%) patients in the PIVS group and 9 (53%) patients in the SSLF group. Overall satisfaction rates were 62% and 76%, respectively. *Conclusion*. The efficacy of PIVS and SSLF is equally poor, and the rate of vaginal erosion is intolerably high with the PIVS method. Based on our study, we cannot recommend the usage of either technique in operative treatment of vaginal vault prolapse.

## 1. Introduction

Posthysterectomy vaginal vault prolapse concerns 0.5–1.8% of all patients who have undergone hysterectomy [1, 2] and 11.6% of the patients with prior hysterectomy for uterine prolapse [2]. Management of vaginal vault prolapse is challenging. The patients are usually elderly, with age-related diseases that decrease their operability. Vaginal approach under regional anesthesia is often preferred to the abdominal approach. Unilateral sacrospinous ligament fixation (SSLF) is a widely accepted vaginal procedure for vaginal vault prolapse treatment. It has a short-term efficacy of up to 96–98% with or without uterine preservation [3, 4], and it provides good long-term objective and subjective outcomes with good cost effectiveness [5]. The most common complications of this procedure are hemorrhages and buttock pain [6].

Traditional operative procedures for the treatment of vaginal vault prolapse are demanding and have rather a long learning curve; that which is why there has been a need to develop optional surgical techniques. The posterior intravaginal slingplasty (PIVS) procedure was presented by Petros in 1997. The advantage of this operation is the easier operative technique with a shorter surgeon's learning curve compared to traditional operative techniques. The efficacy of this technique is acceptable, with success rates varying from 75% to 98% in a short follow-up [7–9]. Mean blood loss and patients' experience of pain have been reported to be minimal [10].

The aim of our retrospective nonrandomized single institutional study was to compare posterior intravaginal slingplasty and unilateral sacrospinous ligament fixation procedures in terms of efficacy, complication rate, and patient satisfaction.

## 2. Material and Methods

Between February 2001 and March 2005, a total of 33 patients with operatively managed vaginal vault prolapse were enrolled in our retrospective study in Oulu University Hospital in Finland. Sixteen patients underwent posterior intravaginal slingplasty operation and 17 unilateral sacrospinous ligament fixation.

The median follow-up time was 16 months (range 6–52). There were no statistically significant differences in age, body mass index, or parity between the study groups (Table 1). All patients in the PIVS group had undergone a prior hysterectomy, while two patients in the SSLF group had a concomitant vaginal hysterectomy, and 15 had previously undergone hysterectomy. Uterine prolapse was indication for prior hysterectomy in 63% and 59% of the cases in the PIVS group and the SSLF group, respectively. In the PIVS group there were two patients who had undergone a prior SSLF operation because of a vaginal vault prolapse.

We used the Pelvic Organ Prolapse Quantification (POP-Q) system to evaluate the objective anatomical outcome. Information on preoperative POP-Q stage was gathered from medical records. The stage of the preoperative prolapse was III or IV in 76% of the cases in the SSLF group and in 50% of the cases in the PIVS group. More detailed information about preoperative symptoms is given in Table 2.

In posterior intravaginal slingplasty technique a polypropylene multifilament tape (IVS Tunneler, Tyco US Surgical, Norwalk, CT, USA; Covidien, Mansfield, MA, USA) is inserted through perineal incisions along the ischiorectal fossae bilaterally towards the incision made in the beginning of the operation at the vaginal apex and pararectal space. The goal of this procedure is to create an artificial uterosacral neoligament for the vaginal vault. 

A sacrospinous ligament fixation procedure was performed by unilateral dissection of the pararectal space on the right side of the patient to expose the sacrospinous ligament, attaching the left and right corners of the vaginal apex with two absorbable sutures to the ligament, using size 0 PDS suture. All the procedures were performed by a single surgeon (M.S.). Intravenous cefuroxime 1.5 g antibiotic prophylaxis was used in all cases.

At the follow-up visit we gathered information about urinary, bowel, and sexual dysfunctions and difficulties using a standardized questionnaire. Overall satisfaction with the treatment was asked. A gynecological examination was performed in horizontal gynecological position, and the maximal size of the prolapse was provoked using the Valsalva maneuver or cough test. Anatomical failure was defined as recurrent prolapse of stage II (any POP-Q point ≥−1) or worse. Apical prolapse failure was defined as point C value ≥ −1. All POP-Q measurements were performed by one person (V.N.).

The data were analyzed using SPSS (v. 17.0) statistical software. Because of the skewed distribution of most variables, we used the Mann-Whitney *U* test. Fisher's exact test was used for the contingency tables of the discrete variables.

## 3. Results

There were no intraoperative bladder, bowel, or vascular injuries. Median intraoperative blood loss was 50 mL (0–350 mL) in both groups. None of the patients required blood transfusion. Detailed information on surgical data is found in Table 2.

Comparison of the most relevant anatomical postoperative outcomes using the POP-Q scale is found in Table 3. We found no significant *P* values between the two study groups concerning these measurements. At the postoperative visit recurrent apical prolapse (point C ≥ −1) was observed in five (31%) patients in the PIVS group and in two (12%) patients in the SSLF group. Anterior vaginal wall prolapse was identified in six patients in the PIVS group; one of these was isolated, and the rest were combined with apical prolapse. In the SSLF group the corresponding figures were five and three. Posterior vaginal wall prolapse was identified in eight patients in the PIVS group and in four patients in the SSLF group; two of these were isolated in the PIVS group and all four in the SSLF group. Altogether postoperative anatomical stage II or worse prolapse was observed in eight (50%) patients in the PIVS group and in nine (53%) patients in the SSLF group at the follow-up visit. Four (33%) anterior wall and five (62%) posterior wall postoperative prolapses were recurrent in the PIVS group. In the SSLF group the figures were four (44%) and three (75%), respectively.

Vaginal erosion of the IVS mesh was observed in four (25%) patients. All of them required surgical removal of the tape and approximation of vaginal edges. One of these patients had a recurrent anterior vaginal wall prolapse which was repaired at the same time with the mesh removal. Four other PIVS patients' prolapse symptoms required reoperation. All of these patients suffered from recurrent apical prolapse; three of them were treated with sacrospinous fixation and one with colpocleisis. Altogether 50% of the PIVS patients required recurrent operative treatment.

In the SSLF group two (12%) patients required a reoperation for recurrent anterior vaginal wall prolapse. No other reoperations were needed during the follow-up.

Eight patients (50%) in the PIVS group and eight patients (47%) in the SSLF group suffered from postoperative urinary symptoms. Symptoms eased off in five (31%) patients in the PIVS group and in ten (59%) patients in the SSLF group (*P* = NS). Six (38%) patients in the PIVS group and eight (47%) patients in the SSLF group suffered from postoperative bowel symptoms.

At the postoperative follow-up visit, 38% of the patients in the PIVS group and 41% in the SSLF group were sexually active. The operation worsened the sexual life of one (6%) patient in the PIVS and of two (12%) patients in the SSLF group (*P* = NS), while improvement was seen in two (13%) and four (24%) patients, respectively. More information about postoperative symptoms and sexual function is found in Table 4.

Ten (62%) patients were satisfied with the operation in the PIVS group and thirteen (76%) in the SSLF group. Half of the unsatisfied patients (4/8) in the PIVS group and all unsatisfied patients (3) in the SSLF group suffered from anatomical failure. One patient in the SSLF group did not have an opinion on overall satisfaction. Statistically significant differences on overall satisfaction were not detected.

## 4. Discussion

Our results demonstrate a nonsignificant difference in the success rate between SSLF and PIVS procedures concerning apical prolapse, 88% versus 69%. Our results are not comparable with other studies where success rates vary from 96% to 100% in SSLF patients and from 82% to 98% in PIVS patients [4, 5, 11–14]. In Jordaan et al.'s study the success rate (75%) for PIVS was close to our results [7].

Half of the patients in both study groups suffered from postoperative prolapse of any compartment. In addition to apical reprolapses also recurrence of anterior and posterior wall prolapses was frequent, from 33% to 75%. A minority of postoperative anterior and posterior vaginal wall prolapses consisted of de novo formation. There are some issues to discuss in explaining this poor result. One of them is our patient material with severe (stages III-IV) preoperative prolapses in 50% in the PIVS group and in 76% in the SSLF group. Preoperative stage IV has been proven to be a risk factor for prolapse recurrence [15, 16]. In addition, the definition of postoperative prolapse differs between various studies. We defined recurrent prolapse in our study as any POP-Q point ≥−1, which is the cut point of POP-Q stage II. The strength in our measurements is that the operative surgeon and the investigator at the postoperative visit were different persons, which rules out surgeon's influence on the POP-Q outcome. 

In our study the rate of reoperation in the PIVS group was 50% during the follow-up compared to 12% in the SSLF group. Previous studies show less need for reoperations in the PIVS patients, 5% to 25% [11–14, 17, 18]. The need for reoperation in SSLF patients has been reported to be 8% which is close to our result [19]. Vaginal erosion rate in the PIVS patients seems to have a direct effect on the need for reoperation. In our study vaginal erosion was found in 25% of the PIVS patients, and all of these erosions were treated operatively. In other studies erosion rate has varied from 0 to 18% [11–14, 17, 18]. 

Postoperative voiding difficulties were common, as nearly half of the patients in both study groups suffered from voiding problems. In de Tayrac et al.'s study [11] only 14% of the PIVS patients and 33% of the SSLF patients had voiding difficulties. Postoperative bowel symptoms were also frequent in our patients. The high percentage of overall postoperative problems is probably a reflection of our study's poor anatomical results. 

Sexual activity among the patients was low. The two most commonly reported reasons for ending of sexual activity were lack of partner or partner's illness. In the PIVS group, two of the three patients with unsatisfying sexual life had dyspareunia. However, the effect of concomitant anterior or posterior vaginal wall surgery on dyspareunia cannot be ruled out. Other studies do not show dyspareunia after PIVS [10, 11], but it has been reported after SSLF [20].

In our study the satisfaction with PIVS was 62% compared to 76% in the SSLF patients. In previous studies, the overall satisfaction measured for PIVS has been 86% [11] and that for SSLF 80% [20]. This difference is actually easy to understand, because our study revealed a high rate of vaginal erosion and need for reoperation in PIVS patients. On the other hand, two thirds of the patients with recurrence in the SSLF group and half of the patients in the PIVS group were satisfied with the result of the operation. This confirms that anatomical result does not directly correlate with patients' symptoms and satisfaction. 

The strength of our study is that all operations were performed by one experienced surgeon with good experience on both operative methods. The limitations of our study are the retrospective approach and the small number of the patients in our study. The main reason for small number of recruited patients is that we abandoned the use of the IVS tape after a short period, because we paid attention to vaginal erosions among PIVS as well as IVS patients [21].

As an idea, posterior IVS is a good one. The intraoperative complication rate is low, which is important when treating elderly patients who are poor candidates for major operations. The amount of mesh material is minimal compared to other meshes intended for the treatment of vaginal vault prolapse. According to the literature, the anatomical result is better than after SSLF [22], and the recovery time is shorter than after abdominal approach. Short-term efficacy has been proven to be acceptable [7–9]. The high incidence of vaginal erosions after usage of IVS tape has given rise to discussion on the reasons for erosion. The microporous and nonelastic nature of the tape has been suggested to be the reason for the vaginal erosion [23]. One recent report demonstrates a significant difference in erosion rate after using two different surgical techniques for the placement of the midurethra IVS tape [24], and the authors suggest that the high erosion rate of the IVS tape is due to surgical technique. However, the use of this multifilament polypropylene tape has diminished over the last few years. Attempts have recently been made to solve the problem of erosion with the use of monofilament polypropylene tape for infracoccygeal sacropexy technique, and the results look promising [14]. 

## 5. Conclusions

The efficacy of SSLF and PIVS techniques is equally poor in the treatment of vaginal vault prolapse. In addition, the need for secondary operation is higher and overall satisfaction is poorer in PIVS patients compared to SSLF patients, mainly due to high incidence of vaginal erosions. The risk for vaginal erosion is significant when using polyfilament tape material, and we have therefore abandoned the use of this material. Based on our study we cannot recommend the use of either technique in treatment of vaginal vault prolapse, and we do not recommend the use of polyfilament tape material with infracoccygeal sacropexy technique.

## Figures and Tables

**Table 1:** Characteristics of the patients.

Characteristics	PIVS (*N* = 16)	SSLF (*N* = 17)
Age (y)	70 (52–80)	62 (48–75)
Body mass index (kg/m^2^)	29 (20–33)	27 (24–39)
Parity	3 (0–7)	3 (1–13)
Previous vaginal hysterectomy	10 (63)	10 (59)
Previous abdominal hysterectomy	3 (19)	3 (18)
Previous laparoscopic hysterectomy	3 (19)	2 (12)
Previous prolapse repair	10 (63)	12 (70)

PIVS: posterior intravaginal slingplasty; SSLF: sacrospinous ligament fixation.

Values are given as median (range) or number (percentage).

**Table 2:** Preoperative symptoms and surgical data.

	PIVS	SSLF
Preoperative symptoms		
Urinary	7 (44)	9 (53)
Bowel	2 (13)	5 (29)
Feeling of the prolapse	10 (62)	8 (47)
Preoperative stage of the prolapse		
II	8 (50)	4 (24)
III	3 (19)	7 (41)
IV	5 (31)	6 (35)
Operative time (min)	61 (40–85)	53 (38–110)
Blood loss (mL)	50 (0–300)	50 (0–350)
Concomitant prolapse surgery	16 (100)	16 (94)
Postoperative hematoma	0	1 (6)
Vaginal erosion	4 (25)	

Values are given as median (range) or number (percentage).

**Table 3:** Postoperative anatomical results.

	PIVS (*N* = 16)	SSLF (*N* = 17)
Point C after surgery	−5.5 (−10–9)	−5 (−8–1)
Tvl after surgery	8 (4–10)	8 (6–10)
Recurrent apical prolapse		
(point C ≥ −1)	5 (31)	2 (12)

PIVS: posterior intravaginal slingplasty; SSLF: sacrospinous ligament fixation; Tvl: total vaginal length.

Point C: cuff in Pelvic Organ Prolapse Quantification (POP-Q) classification (cm from hymen).

Values are given as median (range) or number (percentage).

**Table 4:** Postoperative subjective symptoms and satisfaction.

	PIVS	SSLF
SUI	2 (13)	1 (6)
Urge	2 (13)	5 (29)
Voiding difficulties	7 (44)	8 (47)
Improvement of urinary symptoms	5 (31)	10 (59)
Worsening of urinary symptoms	4 (25)	2 (12)
Improvement of bowel symptoms	3 (19)	4 (24)
Worsening of bowel symptoms	2 (13)	0
Sexual function		
Intercourses	6 (38)	7 (41)
Pain during intercourse	2 (13)	0
Improvement	2 (13)	4 (24)
Worsening	1 (6)	2 (12)
Unsatisfied	3 (62)	13 (76)
Overall satisfaction	10 (62)	13 (76)
Unsatisfied	6 (38)	3 (18)

PIVS: posterior intravaginal slingplasty; SSLF sacrospinous ligament fixation; SUI: stress urinary incontinence.

Values are given as number (percentage).

*P* values were not significant.
